# Description and Validation of a Novel AI Tool, LabelComp, for the Identification of Adverse Event Changes in FDA Labeling

**DOI:** 10.1007/s40264-024-01468-8

**Published:** 2024-07-31

**Authors:** George A. Neyarapally, Leihong Wu, Joshua Xu, Esther H. Zhou, Oanh Dang, Joann Lee, Dharmang Mehta, Rochelle D. Vaughn, Ellen Pinnow, Hong Fang

**Affiliations:** 1 Division of Bioinformatics and Biostatistics, National Center for Toxicological Research (NCTR), US Food and Drug Administration (FDA), Jefferson, AR USA; 2 Office of Scientific Coordination, National Center for Toxicological Research (NCTR), FDA, Jefferson, AR USA; 3 Office of Surveillance and Epidemiology, Center for Drug Evaluation and Research (CDER), FDA, Silver Spring, MD USA

## Abstract

**Introduction:**

The accurate identification and timely updating of adverse reactions in drug labeling are crucial for patient safety and effective drug use. Postmarketing surveillance plays a pivotal role in identifying previously undetected adverse events (AEs) that emerge when a drug is used in broader and more diverse patient populations. However, traditional methods of updating drug labeling with new AE information have been manual, time consuming, and error prone. This paper introduces the LabelComp tool, an innovative artificial intelligence (AI) tool designed to enhance the efficiency and accuracy of postmarketing drug safety surveillance. Utilizing a combination of text analytics and a trained Bidirectional Encoder Representations from Transformers (BERT) model, the LabelComp tool automatically identifies changes in AE terms from updated drug labeling documents.

**Objective:**

Our objective was to create and validate an AI tool with high accuracy that could enable researchers and FDA reviewers to efficiently identify safety-related drug labeling changes.

**Results:**

Our validation study of 87 drug labeling PDF pairs demonstrates the tool's high accuracy, with F1 scores of overall performance ranging from 0.795 to 0.936 across different evaluation tiers and a recall of at least 0.997 with only one missed AE out of 483 total AEs detected, indicating the tool's efficacy in identifying new AEs.

**Conclusion:**

The LabelComp tool can support drug safety surveillance and inform regulatory decision-making. The publication of this tool also aims to encourage further community-driven enhancements, aligning with broader interests in applying AI to advance regulatory science and public health.

**Supplementary Information:**

The online version contains supplementary material available at 10.1007/s40264-024-01468-8.

## Key Points

**Table:** 

LabelComp is an innovative artificial intelligence (AI) tool that automates the identification of changes in AE terms made during updates of drug labeling.
The LabelComp AI tool was validated and demonstrated high accuracy and thus is a fit-for-purpose tool for the identification of AE changes in drug labeling that can enhance regulatory science research and characterization of AE profiles over time.
A human in the loop is necessary for the use of the LabelComp AI tool, as is the case with most other AI tools used for regulatory science research.

## Introduction

The primary purpose of drug labeling is to provide healthcare professionals with a summary of the essential scientific information needed for the safe and effective use of the drug. When a drug is approved, the known adverse reactions associated with it are characterized and included in the drug’s labeling [1], also known as the Full Prescribing Information. Label changes are made when analysis of a drug’s postmarketing experience reveals new, previously undetected adverse reactions [1–4]. Real-world usage after a drug’s approval comprises larger and more diverse patient populations, relative to those in the pre-approval clinical trials. It is important to revise the Full Prescribing Information to provide accurate, up-to-date information on drug safety.

Prior research into safety-related labeling changes has explored the frequency and timing of safety issues and related labeling changes [2–9] and the content of Boxed Warning updates [5]. These studies show that the majority of products have a new safety issue added after approval and these updates continue throughout a drug’s lifecycle [2–4]. Few studies have included characterization of adverse reactions that are incorporated after approval into the various safety-related sections of the Full Prescribing Information [2–4]. These prior efforts to characterize safety issues identified and added after approval entailed a tedious, time-consuming process that involved manually comparing the updated drug labeling with the previous version of labeling. The time to complete the manual review limits the number of products reviewed and frequency of this data abstraction. Specifically, the main challenges are as follows: (i) most drug labeling documents are available in PDF format and need to be specially processed to be machine-readable; (ii) most of the contents of the labeling remain unchanged when there is a labeling change, and many changes do not involve actual adverse event (AE) changes; and (iii) it is time consuming and error prone for reviewers and researchers to manually identify AE changes. Thus, we developed an innovative tool named LabelComp that incorporates artificial intelligence (AI) to assist drug reviewers and researchers in identifying changes to AE terms during labeling updates. The source code for this tool is available at GitHub: https://github.com/seldas/LabelComp. The LabelComp tool supports the comparison of FDA Labeling Documents within a web interface designed to identify product safety labeling changes.

The LabelComp tool has two main components. The first component uses text analytics to extract all texts from the two drug labeling documents (i.e., the current and previous version) that are being compared. The textual differences or updates identified between the drug labeling documents are then highlighted and displayed to facilitate human expert review. The second component leverages AI, in particular a Bidirectional Encoder Representations from Transformers (BERT) model, for natural language processing. The LabelComp tool uses the RxBERT model [10], a domain-specific BERT model developed by our group, which is based on BioBERT [11], with additional training on prescription drug labeling documents curated from FDALabel [12]. RxBERT was applied to detect and highlight AE changes as well as their location within specific label sections (i.e., Boxed Warnings, Contraindications, Warnings and Precautions, Adverse Reactions, and Drug Interactions) in the drug labeling document.

We performed a validation study to determine the accuracy of the LabelComp tool in identifying safety-related labeling changes. Our goal was to create and validate a tool with high accuracy that could enable researchers and FDA reviewers to efficiently identify safety-related labeling changes. If a tool could automate the detection of new AE labeling changes and enable the rapid identification of AE changes in drug labeling, with high recall to avoid missing new AEs added to labeling, this would constitute a significant advancement.

## Materials and Methods

### LabelComp Tool Framework and Tool Development

**Fig. 1:**
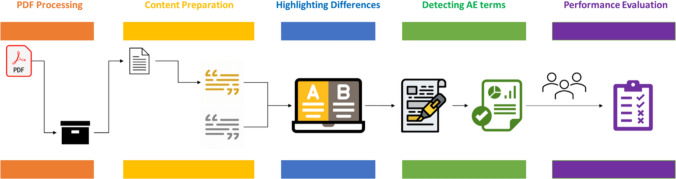
LabelComp tool framework

Figure 1 depicts the LabelComp tool framework. As shown, five main functions were designed for the kernel of the LabelComp tool: (1) PDF file processing; (2) content preparation; (3) highlighting differences; (4) detecting AE terms; and (5) performance evaluation, which entails quality checking, to identify missed AEs or new AEs identified in error.

#### PDF Processing

**Fig. 2:**
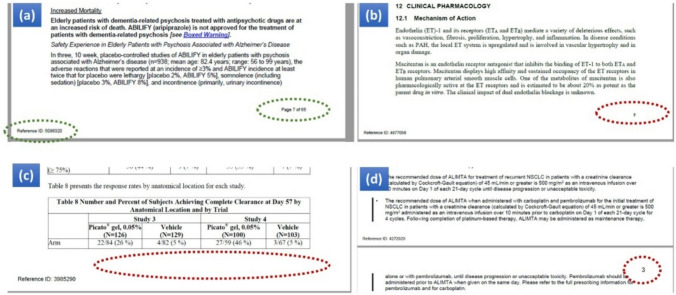
Determination of header and footer for various labeling documents. **a** A typical labeling document should contain the reference ID and page number in the footer. **b** Outlier with a different page number format. **c** Outlier with no page number in footer. **d** Outlier with page number in header

Using PyMuPDF library [13], PDF files were converted into text blocks. A cleaning step was applied to filter out blocks related to header and footer information and to ensure the consistency of the contents. To identify header and footer contents, a mixed strategy was employed: for most labeling documents, a common rule was applied to detect footers which contain the particular words 'Page x of y' and/or 'Reference ID:' (Fig. 2a). However, some PDFs present uncommon patterns and the common rule was not applicable in these cases. For instance, Fig. 2b shows a labeling document using only page numbers instead of 'Page x of y' at the bottom (or on top) of the page. In Fig. 2c, there is no page number in the footer, but the last text block may contain a number, notably extracted from tables or paragraph breaks. In some other cases, the page number was shown at the top instead of the bottom of the page (Fig. 2d). To address these discrepancies, we kept the footer information if it contained uncommon patterns, as reviewers may identify these issues during a secondary check.

**Fig. 3:**
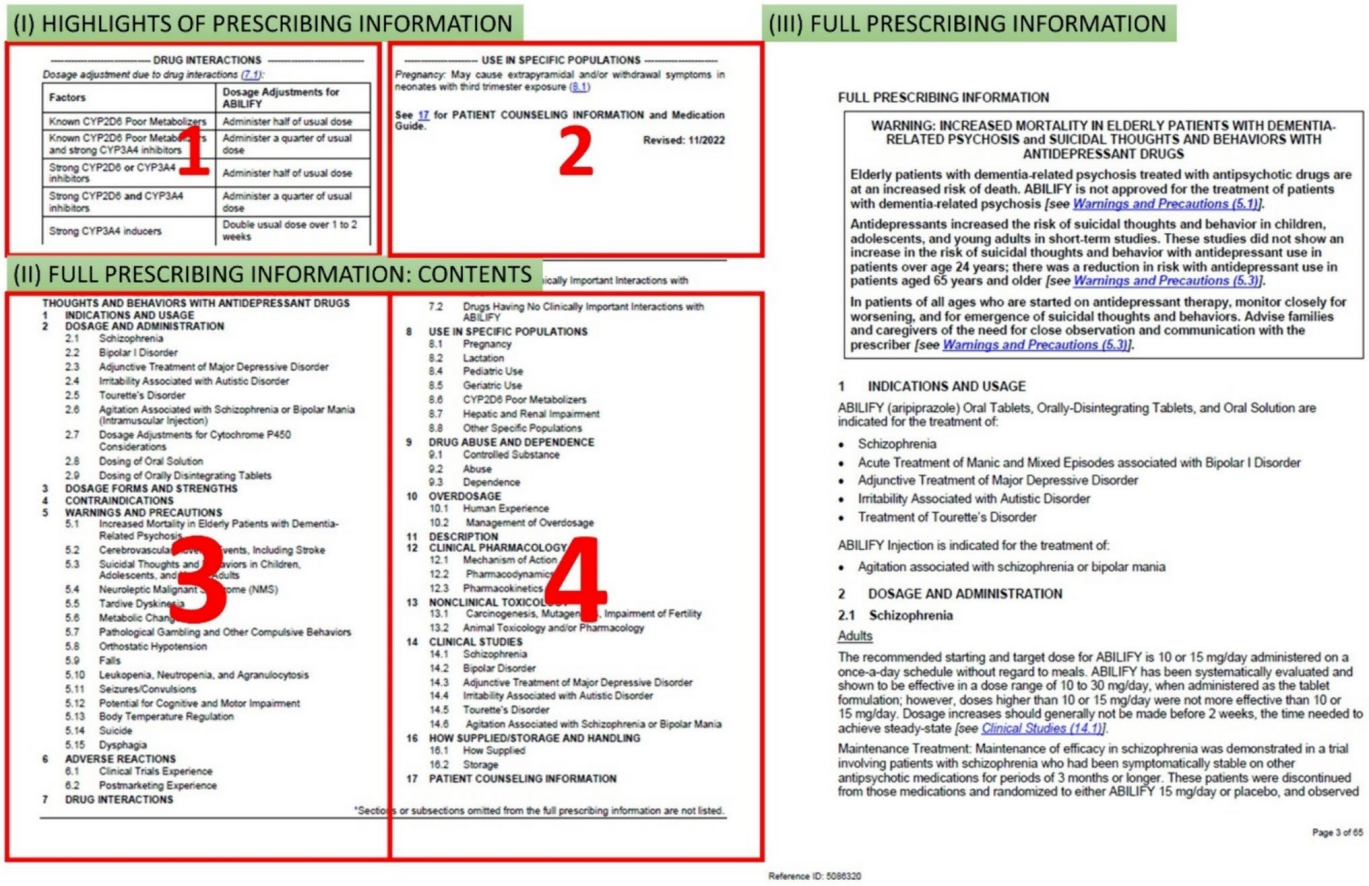
Different layouts in labeling documents: (I) Highlights of Prescribing Information; (II) Full Prescribing Information: Contents; and (III) Full Prescribing Information

For most labeling documents, there are three major components: (i) Highlights of Prescribing Information (highlights), (ii) Full Prescribing Information: Contents (contents), and (iii) Full Prescribing Information (full text). We used pattern matching to recognize content from different components. For highlights and contents sections, the contents were usually organized in a two-column format (Fig. 3), whereas for the full text, the contents were usually organized vertically in a single column. One challenge is that some labeling documents have two different components on the same page. This increased the complexity of dividing these components correctly. For example, by default in a two-column layout, text chunks/blocks are ordered from left to right regardless of the pre-designated sections, though the machine first reads the left-most column in its entirety from top to bottom and then moves to the next column. Therefore, in the absence of section recognition, in Fig. 3 the order of reading text blocks would be 1–3–2–4, where the correct order, based on section headings, should be 1–2–3–4, where 1–2 belongs to the Highlights section, and 3–4 belongs to the Contents section. This example illustrates the importance of recognizing sections in the labeling PDF before processing the content for down-stream analysis.

#### Content Preparation

The full-text component of labeling documents contains the main information of the labeling and is also the most important part that the reviewers will examine. A typical labeling document contains multiple sections, such as Indications and Usage, Warnings and Precautions, Adverse Reactions, etc. In this study, we only focused on the five principal AE-related sections, which are Boxed Warnings; Contraindications (section 4); Warnings and Precautions (section 5); Adverse Reactions (section 6); and Drug Interactions (section 7).

These AE-related sections were extracted by their titles. We used a gradient strategy to match the title patterns as, in most drug labeling, the titles of these sections use a fixed format with the section number, such as ‘^6 ADVERSE REACTIONS’ or ‘^6. ADVERSE REACTIONS’, where ‘^’ means starting a new line. However, there are a few cases that did not follow this rule, such as omission of the section number. In these cases, if they did not match the previous pattern, they were further determined by the pattern ‘^ADVERSE REACTIONS’, where spaces are allowed between the starting of a new line and ‘ADVERSE REACTIONS’.

After extracting the AE-related sections, we further separated each section into sub-sections based on their sub-level title. For example, the ‘Adverse Reactions’ section usually contains subsections like ‘6.1 Clinical Trials Experience’ and ‘6.2 Postmarketing Experience’. The ‘Warnings and Precautions’ section includes the sub-section titles for specific AEs related to this drug.

For sub-section title detection, since each sub-section title usually has a larger font size or is bolded, it would be easy to detect each title if such information could be derived from the PDF file and extracted. However, based on our experience, very few drug labeling PDFs include such information directly. Therefore, we decided to use the regex pattern ‘\.\d+(?=\s[A−Z])’, where ‘\.’ is the escape form of ‘.’, ‘\d+’ means any numbers, ‘\s’ means space, and (?=\s[A−Z]) means the matches of any capitalized character after the pattern to detect the sub-section titles.

#### Highlighting Differences

**Fig. 4:**
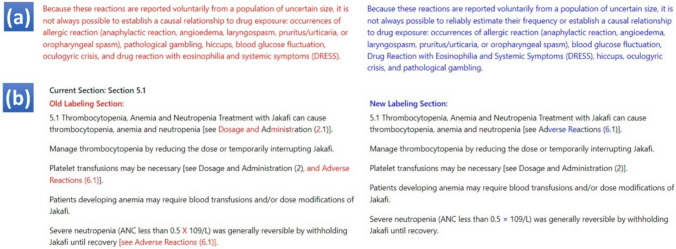
Difference comparison for labeling documents. **a** If too many changes between two sentences are found, the whole sentence will be highlighted; **b** if the content length is small and the changes are countable, the detailed changes will be displayed

After subsections were extracted, they were split into sentences, using the sent_tokenize function under the nltk library [14]. The rationale to split sub-sections into sentences is that the difference-comparison function usually works much better for shorter content (e.g., identify AE terms at the word or sentence level) compared with longer content (e.g., the paragraph or higher level). Thus, if there are many differences with longer content, the difference comparison function will simply label the whole content as different (Fig. 4a), rather than pointing out each difference in detail (Fig. 4b), which is the reviewer-preferred format.

#### Detecting AE Terms

AE terms from the changed text have been detected and recognized by two distinct approaches: (i) RxBERT-based Name Entity Recognition (NER), and (ii) Medical Dictionary for Regulatory Activities (MedDRA^®^)-based pattern matching (keyword). The default option is to use both approaches, which includes AEs identified by either approach.

The RxBERT-based NER approach is developed based on the RxBERT model, a transformer language model originally developed by our team. RxBERT used the pre-trained BioBERT and was further trained with the content of drug labeling documents. In this study, we used the fine-tuned RxBERT model, with supervised training from the AE annotated dataset for the NER task [10]. The F1 score of the RxBERT NER model on two benchmark datasets of TAC2017 and ADE eval is 86.5 and 87.4, respectively, which constituted performance at the top range of current AE detection algorithms from the ADE eval challenge [15].

The MedDRA^®^-based pattern matching approach is based on the regular expression match of MedDRA^®^ low-level terms (LLTs) [16]. Preliminary work has shown that the combination of the trained RxBERT model with the MedDRA^®^ pattern matching approach increases the recall of AE detection more than the MedDRA® pattern matching approach alone. In the current version of LabelComp, the keyword matching process is case-insensitive, which allowed us to detect more AE terms due to the various font formats found in labeling documents. Note that pattern matching may capture some words that are generally not AEs, such as ‘all’, ‘high’, etc. For example, ‘all’ was mostly used in labeling documents as a common word rather than an abbreviation of ‘Acute lymphocytic leukemia’, which has been recorded as a MedDRA^®^ LLT term.

### Validation Study Methods and Statistical Plan

#### Manual Review and Validation

Based on a review of the publicly available ‘Compilation of CDER New Molecular Entity (NME) Drug and New Biologic Approvals’ [17], 87 drugs and therapeutic biologics were approved by the Center for Drug Evaluation and Research (CDER) in 2021 and 2022. At time of the analysis, 60 products had been approved for more than one year and 27 products had been approved for less than one year. Of these, 40/87 had at least one updated labeling posted on Drugs@FDA. This included 37/60 products approved for more than one year and 3/27 approved for less than one year. From the 40 products with labeling changes, there were 52 updated labels posted on Drugs@FDA. The number of updated labels per product ranged from 1 to 5, with most (32/40) having only one labeling update (Appendix A). Product labels (PDFs) were downloaded from Drugs@FDA [18]. The applicable product labels were identified by entering the application number (this is the 6-digit number after ‘NDA’ or ‘BLA’, e.g., NDA021807 for tamoxifen citrate). Reviewers confirmed that the drug name matched the assigned product for the application number searched. Two sequentially approved labels were compared using the LabelComp tool. To compare labels, reviewers clicked on the ‘Compare PDFs’ button. Reviewers then downloaded the results with extracted AE terms in AE-related labeling sections into Excel.

Each reviewer used the tool to determine if the highlighted AE changes, marked with different colors for different categories, were accurately identified by the tool and if these changes were new to that section of the labeling. They had access to the full text of both labeling documents to evaluate the context of the AE changes and to verify if the highlighted text represented actual changes. Reviewers could also compare the text of both labeling documents side by side within the tool to check for any AE changes that the tool might have missed. Additionally, they had the option to review the labeling PDF documents outside of the tool. After using the tool, reviewing the PDF labeling, and consulting the Drug Safety-Related Labeling Changes database [19], reviewers determined if the LabelComp tool missed any newly added issues. For accuracy, reviewers evaluated the LabelComp-identified AEs to determine if the LabelComp tool correctly or incorrectly identified the AE using the following six pre-defined categories: ‘No AE updated (1)’, ‘True new AE (2)’, ‘Not new AE (3)’, ‘Inaccurate (4)’, ‘Irrelevant (5)’, and ‘Missed (6)’. The detailed explanation of these categories is contained in Table 1.

**Table 1:** Categorization of tool findings

Category (number)	Definition
No AE updated (1)	When comparing a pair of PDF labeling documents, the tool did not report a new AE and the reviewer confirmed that there were no AE changes
True new AE (2)	The new AE was correctly identified
Not new AE (3)	The term was already in the previous labeling. This includes terms that may have been picked up due to differences in spelling, formatting, or punctuation
Inaccurate (4)	The AE was partially or incompletely identified by the LabelComp tool
Irrelevant (5)	The term picked up is not an AE. This could include information on the route of administration, instructions for use, drug name, etc.
Missed (6)	The LabelComp tool did not identify an AE

*AE* adverse event

Under comments on accuracy, reviewers were asked to enter information on why the term was incorrectly identified (spelling, punctuation, grammar, spacing, table formatting, same issue-different term, picked up partial word, other information that may be useful). Questions that arose regarding classification were addressed by a designated single reviewer to ensure consistency.

#### Statistical Analysis

The analysis of the information collected was descriptive, describing the accuracy of the LabelComp tool and potential explanations for incorrectly identifying an AE or the tool missing a newly added issue. Among six categories, ‘No AE updated (1)’ was considered correct but was not counted in the confusion matrix. ‘True new AE (2)’ was considered as a true positive (TP); ‘Missed (6)’ was considered as a false negative (FN), ‘Irrelevant (5)’ was considered as a false positive (FP). The other two categories, ‘Not new AE (3)’ and ‘Inaccurate (4),’ did not neatly align with the conventional TP or FP categories. Therefore, we designed tiered evaluation metrics with three tiers, differentiated by the definition of these two categories. In Tier 1, the most tolerant level, both ‘Not new AE’ and ‘Inaccurate’ were counted as a TP; in Tier 2, ‘inaccurate’ is counted as a FP, where ‘Not new AE’ is still considered a TP. In Tier 3, which is the strictest level, both ‘Not new AE’ and ‘Inaccurate’ were counted as a FP (Table 2). The Recall, Precision and F1 score for each tier were then calculated respectively (Formula 1). Among these metrics, recall is the most important metric as it is necessary to try to capture all new AEs added to the labeling without missing any; otherwise, labeling must be reviewed manually to try to identify any AEs that may have been missed which is highly burdensome, time consuming, and unscalable.

**Table 2:** Definition of the tiered evaluation

Category	Tier 1	Tier 2	Tier 3	Descriptive example
No AE updated (1)	N/A	N/A	N/A	Compared two versions of PDF labeling. No new AEs identified. The tool correctly ‘did not identify’ any new AEs as the changes appear to have entailed deletions in section 6.1 versus new AEs
True new AE (2)	TP	TP	TP	The tool reported a new AE term, ‘chest discomfort,’ and it was confirmed by the reviewer
Not new AE (3)	TP	TP	FP	The tool reported a new AE term, ‘splenomegaly cholelithiasis dehydration fracture.’ The tool reported this as a combination of four separated AE terms and all of these terms were already included in the old version
Inaccurate (4)	TP	FP	FP	The tool reported a new AE term, ‘pressure,’ along with ‘blood pressure.’ The reviewer confirmed that the former AE term was inaccurately reported since it was part of the latter report term
Irrelevant (5)	FP	FP	FP	The tool reported a new AE term, ‘cancer.’ The reviewer confirmed it is irrelevant to AE term since cancer was mentioned in the context that related to those studies with cancer patients
Missed (6)	FN	FN	FN	The tool missed reporting an AE term, ‘abscess.’ The reviewer found this AE during a second-hand examination of the different texts between two PDFs

1 $$\text{Recall}=\frac{\text{TP}}{\text{TP}+\text{FN}};\text{ Precision}=\frac{\text{TP}}{\text{TP}+\text{FP}};\;F1=2*\frac{\text{Recall}*\text{Precision}}{\text{Recall}+\text{Precision}}$$

## Results and Discussion

### Highlighting the Differences Between Two Labeling Documents

**Fig. 5:**
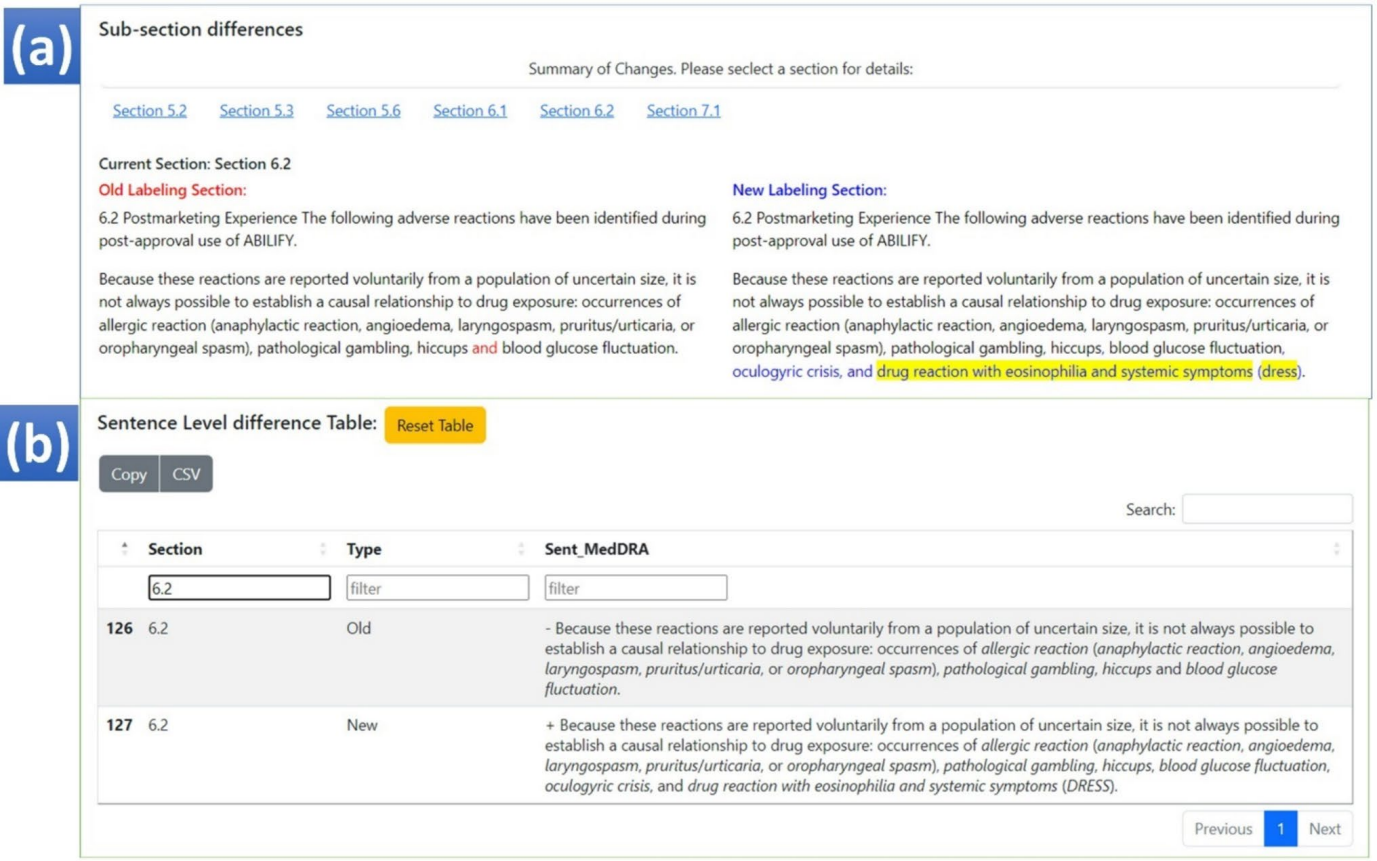
Two styles of visualization to show differences. **a** Column-wise comparison; **b** The summary table

Two visualization approaches were used to present the differences between two PDF documents. First, we developed a column-wise comparison interface to compare every sentence in a sub-section (Fig. 5a). For the example shown in Fig. 5a, we can see that several new terms are added in the new labeling document showing on the right side (highlighted in blue font [not identified by the tool as an AE term] with yellow highlighting for the tool-identified AE terms). Also, we provided the whole sentence records in a summary table (Fig. 5b) for batch analysis. The whole table can be exported as a .csv file or directly in the clipboard.

### AE Term Recognition

**Fig. 6:**
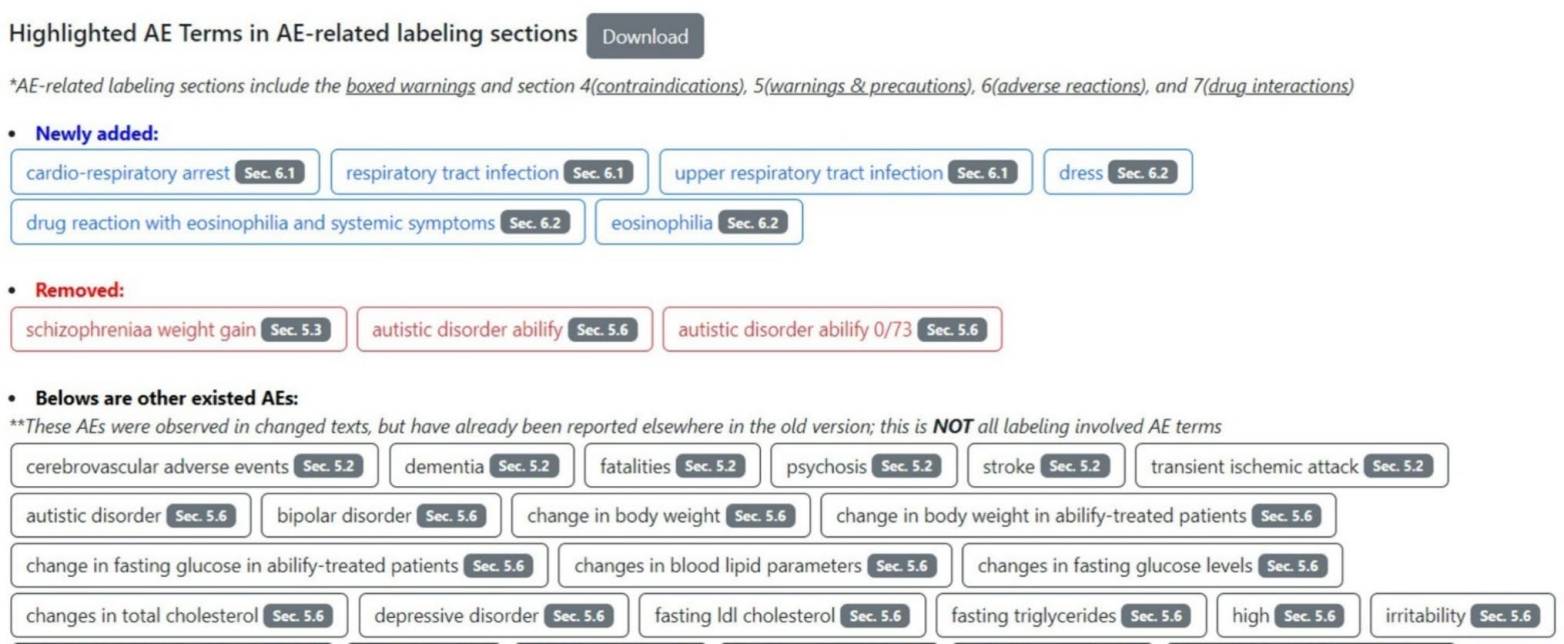
Detected AEs were placed into three categories, as newly added, removed, and pre-existing AEs in the tool result page, respectively

After the differences between two labeling documents are identified, we further detected AE terms from different parts of the documents. The detected AEs, based on their occurrence in both older and newer labeling documents, can be categorized into three types: (a) newly added; (b) removed; and (c) already existing AEs (Fig. 6). In this manuscript, we focused on newly added AEs which were only found in the new labeling document, but not found in the same major section of the old labeling document. For example, if the term cardio-respiratory arrest was found in section 6.1 of the new labeling document, and it did not exist in the whole section 6 (Adverse Reactions) of the old labeling document, it would be listed as ‘newly added’ in blue font.

### Performance Evaluation

The output of the LabelComp tool was assessed by each reviewer’s manual evaluation, as depicted in Fig. 1 as performance evaluation. To assess the performance of the LabelComp tool in recognizing AE changes, we examined its identification of newly added terms and its accuracy in differentiating between new and existing AEs or misidentifying non-AE terms. For analysis, occurrences of AE terms in the new labeling document were counted only once if repeated.

As the result, 87 drug labeling PDF pairs were validated by the reviewers, including 21 PDF pairs without any identified newly added AE terms. For the remaining 66 PDF pairs, there were in total 462 AEs identified by the tool, consisting of the remaining five categories defined in three tiers. The original count of all categories, and tiered recall, precision, and F1 score were measured as shown in Table 3.

**Table 3:** LabelComp tool validation results

Findings	Category	Counts	Statistics (used category)	Tier 1 (used category)	Tier 2 (used category)	Tier 3 (used category)
Total		483	Total (2–6)	462	462	462
No AE updated	1	21	TP	407 (2–4)	332 (2, 3)	305 (2)
True new AE	2	305	FP	54 (5)	129 (4, 5)	156 (3–5)
Not new AE	3	27	FN (6)	1	1	1
Inaccurate	4	75	Precision	0.881	0.720	0.661
Irrelevant	5	54	Recall	0.997	0.997	0.997
Missed	6	1	F1 score	0.936	0.836	0.795

*AE* adverse event, *FN* false negative, *FP* false positive, *TP* true positive

We observed that, overall, LabelComp achieved high statistical performance as the Tier 1 performance yielded an F1 score of 0.936 and Tier 3 performance yielded an F1 score of 0.795. Particularly, the high recall of 0.997 indicates that LabelComp can identify most newly added AEs as it only missed one AE that was found by a human. To further support our findings, one of the authors (RDV) conducted a post-hoc, additional validation analysis, akin to a sensitivity analysis. This entailed assessing, validating, and characterizing LabelComp tool findings and errors by reviewing labels from 53 drugs approved by CDER in 2020. This analysis generated similar F1 scores across the three tiers (data not shown) and a recall of 1 as no newly added AEs were missed. Thus, the additional analysis confirmed our findings that the tool performs well with a high recall.

Our validation study found that the LabelComp tool is highly accurate with a high recall of at least 0.997 across the three tiers, F1 scores ranging from 0.795 to 0.936 across tiers, and precision ranging from 0.661 to 0.881 across tiers. As recall is the most important metric for the identification of new AEs added to the label, this demonstrates that this tool is highly effective and fit for purpose. Missing one or two AEs in an extensive set of labeling documents will likely not substantially affect the efficiency gain achieved by this tool. In this case, the one missed AE was in a footnote of a table on adverse reactions. Also, there is rarely an AI or other tool that has perfect sensitivity, and it may not be feasible to develop one. The additional ad-hoc validation by a separate reviewer confirmed the high sensitivity of the tool, so there is good confidence based on the initial, independent validations that it is highly effective.

## Conclusion

This study introduced LabelComp, an AI-driven tool designed to identify adverse event (AE) changes in drug labeling documents. Leveraging advanced AI techniques, including the RxBERT model for natural language processing, LabelComp effectively addresses the challenges of manual labeling comparison.

Our validation study demonstrated LabelComp’s high accuracy in detecting new AEs in drug labeling, achieving a recall rate of 0.997, whereby nearly all newly added AEs are detected. A number of the authors have conducted manual reviews of labels to identify AEs before LabelComp was developed and anecdotally found that the tool saved a lot of time vis a vis their prior manual reviews of labels. The tool has the potential to significantly reduce the time required for reviewers to identify true positive AE changes compared with manual methods, enhance the efficiency of FDA’s regulatory review, and advance regulatory science research.

As an open-source tool, LabelComp allows researchers to use, improve, and share enhancements with the public. Parts of the code could be adapted to support other review or regulatory science activities. For example, the ability to convert a PDF drug labeling document into machine-processable text and the ability to identify AEs in document sections are valuable functions that could be applied to other uses. Limitations include (i) not capturing all AE changes (missed only 1 out of 462 total AEs); (ii) identifying AEs that do not represent actual changes; (iii) only undergoing initial FDA validation; and (iv) requiring human validation to ensure quality, as with most AI tools in regulatory research.

LabelComp’s ability to automate AE identification and provide detailed comparison outputs is a significant contribution to pharmacovigilance. Building on the work with RxBERT and LabelComp, the FDA will continue to develop and iterate AI tools for drug safety review and research, consistent with its broader mission to use AI and emerging scientific approaches to advance regulatory science and public health [20].

## Supplementary Information

Below is the link to the electronic supplementary material.

Supplementary file1 (PDF 137 KB)

## Declarations

### Data availability

For the validation study, the labeling data is publicly available at: https://www.accessdata.fda.gov/scripts/cder/daf/index.cfm.

### Code availability

The source code of the LabelComp tool is available at https://github.com/seldas/labelComp/.
