# Supplementary material for: Description and Validation of a Novel AI Tool, LabelComp, for the Identification of Adverse Event Changes in FDA Labeling
Source: Drug Saf. 2024 Jul 31;47(12):1265–74. doi: 10.1007/s40264-024-01468-8 (PMC11554693; doi:10.1007/s40264-024-01468-8)
Supplement: Supplementary file 1 — Supplementary file1 (PDF 137 KB) [file 40264_2024_1468_MOESM1_ESM.pdf]

Appendix A. Validation study: drug labeling changes

Description and validation of a novel AI tool, LabelComp, for the identification of adverse event changes in FDA labeling

Drug Safety

George A. Neyarapally, Leihong Wu, Joshua Xu, Esther H. Zhou, Oanh Dang, Joann Lee, Dharmang Mehta, Rochelle D. Vaughn, Ellen Pinnow, Hong Fang

Author Affiliations:

George A. Neyarapally, PharmD, JD, MPH, Esther Zhou, MD, PhD, Oanh Dang, PharmD, Joann H. Lee, PharmD, Dharmang Mehta, PharmD, Ellen Pinnow, PhD, MS, Rochelle D. Vaughn, MD, MS: Office of Surveillance and Epidemiology, Center for Drug Evaluation and Research, FDA, Silver Spring, MD, USA.

Leihong Wu, PhD and Joshua Xu, PhD: Division of Bioinformatics and Biostatistics, National Center for Toxicological Research (NCTR), US Food and Drug Administration (FDA), Jefferson, Arkansas, USA.

Hong Fang, PhD: Office of Scientific Coordination, National Center for Toxicological Research (NCTR), FDA, Jefferson, Arkansas, USA.

Email address of corresponding author: George.neyarapally@fda.hhs.gov

Proprietary Name	Active Ingredient/Moiety	NDA or BLA	Application Number	FDA Approval Date	Label Change Action Date(s)				
Verquvo	vericiguat	NDA	214377	1/19/2021	05/10/2023				
Cabenuva	cabotegravir; rilpivirine (co-packaged)	NDA	212888	1/21/2021	1/31/2022	2/7/2022	3/23/2022	3/29/2022	2/27/2023
Tepmetko	tepotinib	NDA	214096	2/3/2021	3/23/2023				
Evkeeza	evinacumab-dgnb	BLA	761181	2/11/2021	3/21/2023				
Cosela	trilaciclib	NDA	214200	2/12/2021	4/5/2023				
Amondys 45	casimersen	NDA	213026	2/25/2021	3/23/2023				
Nulibry	fosdenopterin	NDA	214018	2/26/2021	10/27/2022				
Ponvory	ponesimod	NDA	213498	3/18/2021	10/28/2021				
Qelbree	viloxazine	NDA	211964	4/2/2021	4/29/2022				
Nextstellis	drospirenone and estetrol	NDA	214154	4/15/2021	4/29/2022				
Jemperli	dostarlimab-gxly	BLA	761174	4/22/2021	4/28/2022	2/9/2023			
Zynlonta	loncastuximab tesirine-lpyl	BLA	761196	4/23/2021	10/12/2022				

Proprietary Name	Active Ingredient/Moiety	NDA or BLA	Application Number	FDA Approval Date	Label Change Action Date(s)				
Empaveli	pegcetacoplan	NDA	215014	5/14/2021	2/8/2023				
Rybrevant	amivantamab-vmjw	BLA	761210	5/21/2021	11/4/2022				
Lumakras	sotorasib	NDA	214665	5/28/2021	11/21/2022	1/20/2023			
Brexafemme	ibrexafungerp	NDA	214900	6/1/2021	6/15/2022				
Aduhelm	aducanumab-avwa	BLA	761178	6/7/2021	7/7/2021	4/29/2022	2/8/2023		
Rylaze	asparaginase erwinia chrysanthemi (recombinant)-rywn	BLA	761179	6/30/2021	11/18/2022				
Kerendia	finerenone	NDA	215341	7/9/2021	9/1/2022				
[drug marketed without a proprietary name]	fexinidazole	NDA	214429	7/16/2021	12/15/2021				
Bylvay	odevixibat	NDA	215498	7/20/2021	10/21/2022	6/13/2023			
Skytrofa	lonapegsomatropin-tcgd	BLA	761177	8/25/2021	10/20/2022				
Exkivity	mobocertinib	NDA	215310	9/15/2021	3/2/2023				
Qulipta	atogepant	NDA	215206	9/28/2021	4/17/2023				
Livmarli	maralixibat	NDA	214662	9/29/2021	4/1/2022	3/13/2023			
Tavneos	avacopan	NDA	214487	10/7/2021	7/6/2022				
Scemblix	asciminib	NDA	215358	10/29/2021	6/26/2023				
Livtencity	maribavir	NDA	215596	11/23/2021	9/8/2022	4/26/2023			
Cytalux	pafolacianine	NDA	214907	11/29/2021	12/16/2022				
Tezspire	tezepelumab-ekko	BLA	761224	12/17/2021	2/1/2023				
Leqvio	inclisiran	NDA	214012	12/22/2021	7/7/2023				
Quviviq	daridorexant	NDA	214985	1/7/2022	11/8/2022				
Cibinqo	abrocitinib	NDA	213871	1/14/2022	2/9/2023				
Kimmtrak	tebentafusp-tebn	BLA	761228	1/25/2022	11/29/2022				
Vabysmo	faricimab-svoa	BLA	761235	1/28/2022	1/27/2023				
Enjaymo	sutimlimab-jome	BLA	761164	2/4/2022	1/25/2023				
Ztalmy	ganaxolone	NDA	215904	3/18/2022	11/9/2022	06/21/2023			
Amvuttra	vutrisiran	NDA	215515	6/13/2022	2/16/2023				
Terlivaz	terlipressin	NDA	22231	9/14/2022	1/9/2023				
Imjudo	tremelimumab-actl	BLA	761289	10/21/2022	10/28/2022				
